# Reply to ‘Revisiting postsurgical aortic pseudoaneurysm mortality and treatment options’

**DOI:** 10.1007/s12471-024-01882-8

**Published:** 2024-07-08

**Authors:** Romy R. M. J. J. Hegeman, Martin J. Swaans, Jurriën M. ten Berg, Patrick Klein

**Affiliations:** 1 Present Address: Department of Cardiothoracic Surgery, Amsterdam UMC, Amsterdam, The Netherlands; 2 Department of Cardiology, St. Antonius Hospital, Nieuwegein, The Netherlands; 3 Department of Cardiology, Maastricht UMC+, Maastricht, The Netherlands; 4 Department of Cardiothoracic Surgery, St. Antonius Hospital, Nieuwegein, The Netherlands

We appreciate Mr Bervoets’ interest [1] in our recent publication in the *Netherlands Heart Journal* [2]. However, the key point of our study design is overlooked. This is a case series, which is defined by observations made on a series of individuals, who usually all receive the same intervention; there is no control group. We describe our single-centre experience with one potential treatment option, which is not a systematic review of all available treatment options, nor a state-of-the-art review of the ultimate treatment strategy.

To this date, the European Society of Cardiology Guidelines on the diagnosis and treatment of aortic diseases state that in patients with aortic pseudoaneurysms, interventional or open surgical interventions are always indicated if feasible and independently of size [3]. Evidence supporting this conclusion in the guidelines does remain scarce, but we would like to underscore the risk of conservative treatment.

Matsuzawa et al. indeed discuss a mortality rate of 61% for non-surgical treatment owing to a relatively high incidence of rupture [4], but we acknowledge that this originates from a study by Mulder et al. on mortality associated with pseudoaneurysms following aortoiliac and aortofemoral surgery [5]. We therefore consider this a corrigendum to our original article. Although this incidence can theoretically not be generalised to thoracic aortic surgery, the mechanism of potentially fatal rupture remains similar.

## References

[1] Bervoets B (2024). Revisiting postsurgical aortic pseudoaneurysm mortality and treatment options. Neth Heart J.

[2] Hegeman RRMJJ (2023). Transcatheter closure of postsurgical aortic pseudoaneurysms guided by three-dimensional image reconstruction: a&nbsp;single-centre experience. Neth Heart J.

[3] Erbel R, et al. ESC Guidelines on the diagnosis and treatment of aortic diseases: Document covering acute and chronic aortic diseases of the thoracic and abdominal aorta of the adult. Eur Heart J. 2014;2014. 10.1093/eurheartj/ehu281 25173340

[4] Matsuzawa T (2021). Successful endovascular treatment of an ascending aortic pseudoaneurysm using an Amplatzer Vascular Plug II. Gen Thorac Cardiovasc Surg.

[5] Mulder EJ (1998). Morbidity and mortality of reconstructive surgery of noninfected false aneurysms detected long after aortic prosthetic reconstruction. Arch Surg.

